# Obesity promotes 7,12-dimethylbenz(*a*)anthracene-induced mammary tumor development in female zucker rats

**DOI:** 10.1186/bcr1263

**Published:** 2005-06-06

**Authors:** Reza Hakkak, Andy W Holley, Stewart L MacLeod, Pippa M Simpson, George J Fuchs, Chan Hee Jo, Thomas Kieber-Emmons, Soheila Korourian

**Affiliations:** 1 Department of Dietetics and Nutrition, University of Arkansas for Medical Sciences, Little Rock, Arkansas, USA; 2 Department of Pediatrics, University of Arkansas for Medical Sciences, Little Rock, Arkansas, USA; 3 Arkansas Children's Hospital Research Institute, Little Rock, Arkansas, USA; 4 Arkansas Cancer Research Center, University of Arkansas for Medical Sciences, Little Rock, Arkansas, USA; 5 Department of Pathology, University of Arkansas for Medical Sciences, Little Rock, Arkansas, USA

## Abstract

**Introduction:**

High body mass index has been associated with increased risk for various cancers, including breast cancer. Here we describe studies using 7,12-dimethylbenz(*a*)anthracene (DMBA) to investigate the role of obesity in DMBA-induced mammary tumor susceptibility in the female Zucker rat (*fa/fa*), which is the most widely used rat model of genetic obesity.

**Method:**

Fifty-day-old female obese (*n *= 25) and lean (*n *= 28) Zucker rats were orally gavaged with 65 mg/kg DMBA. Rats were weighed and palpated twice weekly for detection of mammary tumors. Rats were killed 139 days after DMBA treatment.

**Results:**

The first mammary tumor was detected in the obese group at 49 days after DMBA treatment, as compared with 86 days in the lean group (*P *< 0.001). The median tumor-free time was significantly lower in the obese group (*P *< 0.001). Using the days after DMBA treatment at which 25% of the rats had developed mammary tumors as the marker of tumor latency, the obese group had a significantly shorter latency period (66 days) than did the lean group (118 days). At the end of the study, obese rats had developed a significantly (*P *< 0.001) greater mammary tumor incidence (68% versus 32%) compared with the lean group. The tumor histology of the mammary tumors revealed that obesity was associated with a significant (*P *< 0.05) increase in the number of rats with at least one invasive ductal and lobular carcinoma compared with lean rats.

**Conclusion:**

Our results indicate that obesity increases the susceptibility of female Zucker rats to DMBA-induced mammary tumors, further supporting the hypothesis that obesity and some of its mediators play a significant role in carcinogenesis.

## Introduction

Obesity has been identified as an epidemic in the USA for more than two decades, yet the proportion of overweight and obese adults in the population continues to grow. The most recent data from the 1999–2000 National Health and Nutrition Examination Survey (NHANES) [1] showed that almost 65% of adults in the USA are overweight, defined as having a body mass index (BMI) greater than 25 kg/m^2^. This is a significant increase from the 56% for adults reported as overweight in NHANES III, which was conducted between 1988 and 1994. The prevalence of obesity, defined as a BMI of 30 kg/m^2 ^or greater, also increased dramatically from 23% to 31% during the same period. It is estimated that the prevalence of obesity in adults will rise to 39% by the year 2008. This trend has alarming health and economic implications, because obesity is associated with major causes of morbidity and mortality such as diabetes, cardiovascular disease and several types of cancers, including breast cancer [2,3]. Breast cancer is the most common malignant tumor among women, being the second leading killer of women in the USA [4]. Epidemiological studies link breast cancer and obesity in postmenopausal women [4-6]; almost half of breast cancer cases among postmenopausal women occur in those with a BMI in excess of 29 kg/m^2 ^[7]. Although a number of studies have shown that excess weight is a risk factor for breast and other cancers, Calle and coworkers [8] concluded that 20% of all deaths from cancer in women aged over 50 years old could be attributed to being overweight or obese.

In experimental models, higher body weight has been associated with an increase in both spontaneous and chemically induced mammary tumors in various strains of mice [7,9-12]. In order to understand better the mechanisms that are associated with obesity and cancer, we have turned our attention to Zucker rats as a model. The Zucker rat (*fa/fa*) is the best known, most widely used rat model of genetic obesity. Obesity in the Zucker rat is inherited as an autosomal-recessive trait caused by a mutation (*fa*) in the leptin receptor gene [13,14], discovered by Zucker and Zucker [15,16]. Animals homozygous for the *fa *allele become noticeably obese by age 3–5 weeks, and by 14 weeks of age more than 40% of their body is composed of lipids [17]. Many investigators have used this model to study the development, etiology, associated pathogenesis, possible treatment, and putative mechanisms of severe obesity [18]. Obese Zucker rats develop hyperinsulinemia and insulin resistance before they develop obesity-associated, non-insulin-dependent diabetes mellitus in a manner similar to that in humans [19]. Lean Zucker rats, in contrast, exhibit normal metabolic function and are considered ideal controls. Consequently, this model is an ideal one in which to investigate the relationship between obesity and mammary tumor development.

There has been only one study published that used female Zucker rats as a model to investigate the role of obesity in mammary tumor development [20]. That study used *N*-methyl-*N*-nitrosourea (MNU) as a carcinogen and reported that lean rats developed more mammary tumors than did obese ones. Because of its similarity to human breast cancer, researchers have widely used the 7,12-dimethylbenz(*a*)anthracene (DMBA)-induced rat mammary carcinoma model to investigate breast carcinogenesis. In the present study we observed that DMBA-induced mammary tumors in obese Zucker rats develop faster than they do in lean counterparts, and that obese animals were at more than double the risk for developing DMBA-induced mammary tumors. Therefore, our results suggest that this model parallels the epidemiological data and is an appropriate model in which to investigate the mechanism(s) that underlie the role of obesity in mammary tumor development and possible prevention strategies.

## Materials and methods

### Experimental design

All animal protocols were approved by the Institutional Animal Care and Use Committee at the University of Arkansas for Medical Sciences. Obese *fa/fa *(*n *= 25) and lean (*n *= 28) Zucker rats were purchased at age 6 weeks (Harlan Industries, Indianapolis, IN, USA) and housed in the animal facilities at the Arkansas Children's Hospital Research Institute. Rats were housed two per cage in polycarbonate cages and allowed free access to water and regular chow (Harlan-Teklad, Madison, WI, USA). At the age of 50 days, all rats received via gavage 65 mg/kg DMBA (Sigma Chemical Co., St. Louis, MO, USA), a chemical procarcinogen used widely to produce mammary adenocarcinoma in rats, in sesame oil [21,22]. Rats were weighed twice per week. Beginning 2 weeks after DMBA treatment, all rats were palpated twice weekly to detect mammary tumors. The detection date and location of each mammary tumor was recorded for each rat. Rats were killed 139 days after DMBA treatment. All mammary tumors were excised, counted, and weighed. Rats with tumor masses exceeding 2.5 cm in diameter were killed early for humane reasons, in accordance with our Institutional Animal Care and Use Committee-approved animal protocol. Sections of all tumors were placed in 10% neutral buffered formalin for histopathologic analysis. Sections (5 μm) of the paraffin-embedded tumors were stained with hematoxylin and eosin for histologic analysis.

### Pathology

A board-certified anatomic pathologist (SK) evaluated tumors in a blinded protocol and classified them as benign or intraductal proliferation, as shown by multiple papillomas or with ductal hyperplasia, ductal carcinoma *in situ*, or invasive ductal and lobular carcinoma.

### Statistical analysis

A one-way analysis of variance [23] was used to analyze body weight following DMBA treatment. The tumor-free times were plotted using a Kaplan–Meier curve [24] and the median times were compared using a generalized Wilcoxon test [25]. When there is no censoring, this test is equivalent to a Mann–Whitney rank sum test. Fisher's exact test [26] was used to compare the percentage of rats with tumors and tumor histology in each group. The median numbers of tumors per tumor-bearing rat (multiplicity) for each group were compared using the nonparametric Mann–Whitney test [27]. Statistical significance was set at *P *< 0.05, and all *P *values were unadjusted for multiple comparisons. For the few rats that were killed early because of tumor burden, we assumed that the number of tumors remained constant until the end of the study. Data analyses were generated and plots were constructed using SPSS^© ^version 12.0 for Windows (SPSS Inc., Chicago, IL, USA).

## Results

### Body and organ weights

**Figure 1:**
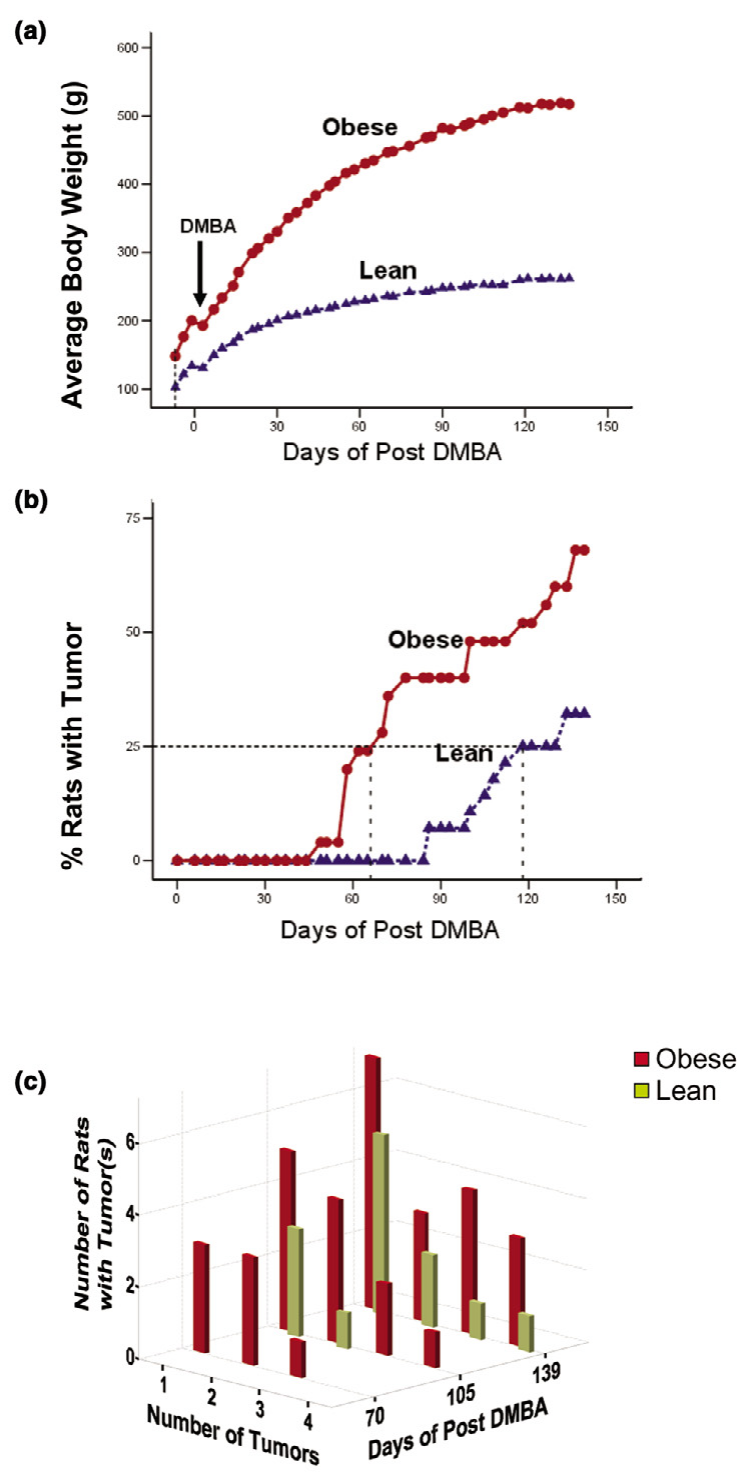
Body weights and mammary tumor incidence and multiplicity in DMBA-treated Zucker rats. **(a) **Body weights of lean and obese female rats. **(b) **Mammary tumor incidence (percentage of rats with tumors) in female rats. Dashed lines indicate the post-DMBA (7,12-dimethylbenz(*a*)anthracene) days at which 25% of the obese and lean rats developed at least one mammary tumor. **(c) **Mammary tumor multiplicity in obese and lean rats.

As expected, all rats gained weight during the course of the experiment. The average body weights (mean ± standard error) are shown in Fig. 1a. Obese rats gained significantly (*P *< 0.001) more weight than did lean rats. At the beginning of the study (7 days before DMBA treatment) the mean body weights of lean and obese rats were 102.5 ± 1.5 g and 148 ± 3.5 g, respectively. At the end of the study, the final mean body weights for lean and obese rats were 262.3 ± 4.2 g and 517.2 ± 17.9 g, respectively. The obese rats gained approximately twice the weight of the lean rats. Obesity was associated with a significant increase in liver (*P *< 0.001) and kidney (*P *< 0.001) weights compared with those in the lean group (Table 1). This increase in liver weight in obese rats also was evident when liver weight was expressed as percentage of body weight (*P *< 0.001); however, kidney weight as a percentage of body weight was not affected significantly by obesity.

### Time course for tumor formation, latency and multiplicity

The time course of palpable mammary tumor detection is shown in Fig. 1b, and data are presented in Table 2. The tumor latency (the number of days after DMBA treatment until detection of the first mammary tumor) was shorter in obese rats than in the lean rats. The first mammary tumor detected in obese rats was 49 days after DMBA treatment versus 86 days in lean rats, which represents a significant 37-day delay for the development of mammary tumors in lean rats (Table 2). In addition, it took only 66 days after DMBA treatment for 25% of the obese rats to develop mammary tumors versus 118 days in lean rats – a delay of 52 days. The median tumor-free time was significantly lower in the obese group (*P *< 0.001; Fig. 1b). By the end of the study (139 days after DMBA treatment), 68% of obese rats had developed mammary tumors as compared with only 32% of the lean rats (*P *< 0.001; Fig. 1b). The median number of mammary tumors per tumor-bearing rat (multiplicity) increased from one tumor per rat in the lean group (range: one to four tumors per rat) to two tumors per rat in the obese group (range: one to four tumors per rat; Table 2).

For further comparison of mammary tumor development we evaluated tumor multiplicity at three time points (70, 105 and 139 days after DMBA treatment). As demonstrated in Fig. 1c, the obese rats developed mammary tumors earlier than did lean rats. At 70 days after DMBA treatment, several rats in the obese group had one to three tumors each, whereas no mammary tumors were detected in the lean group. At 105 days after DMBA treatment there were several rats in the obese group with tumors, including five rats with one tumor each, four rats with two tumors, two rats with three tumors, and one rat with four tumors. This is in contrast to the lean group, in which only three rats had one tumor each and one rat had two tumors. At 139 days after DMBA treatment, there were seven rats in obese group with one tumor each, three rats with two tumors, four rats with three tumors, and three rats with four tumors. In contrast, the lean group had five rats with one tumor each, two rats with two tumors, one rat with three tumors, and one rat with four tumors. These findings indicate for the first time that obese Zucker rats are an excellent model for investigating the role of obesity in DMBA-induced mammary tumor development.

### Mammary tumor characteristics

**Figure 2:**
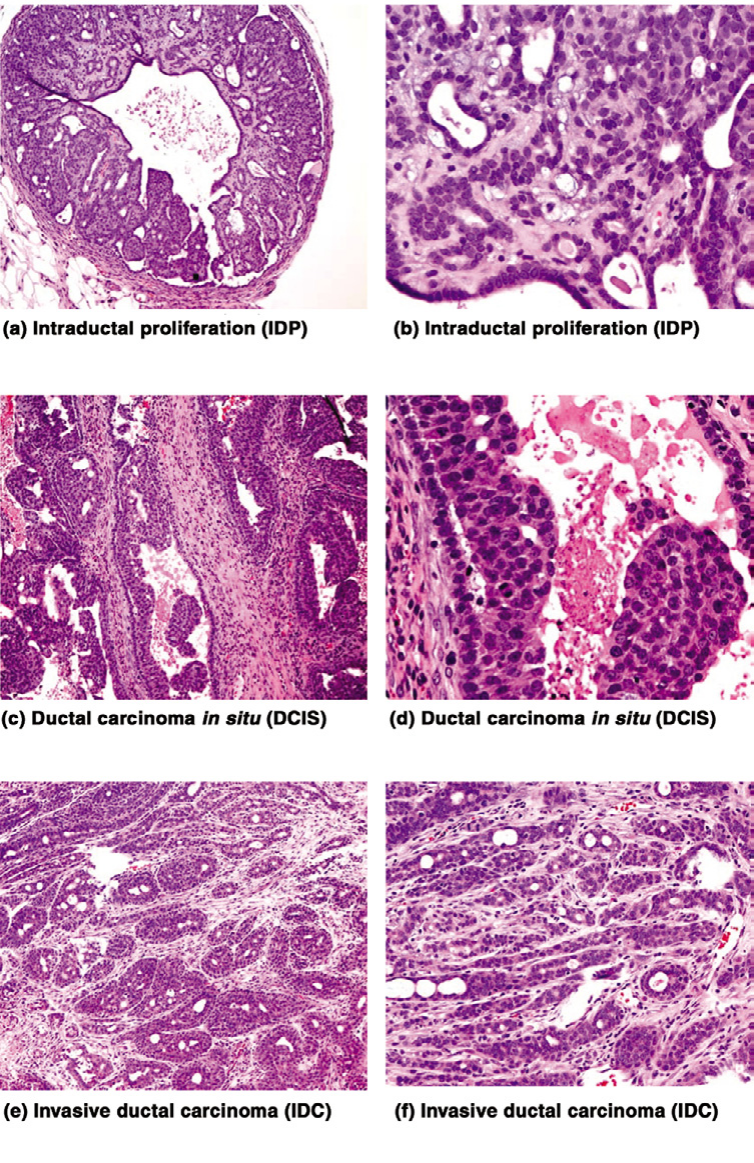
Mammary tumor histology. **(a) **Intraductal proliferation (intraductal papilloma). Original magnification: 40×. **(b) **Higher magnification of panel a. Original magnification 200×. **(c) **Ductal carcinoma *in situ*. Original magnification: 40×. **(d) **Higher magnification shows proliferation of uniform neoplastic cells with high nucleus to cytoplasmic ratio within ductal structures. Original magnification: 200×. **(e) **Invasive ductal carcinoma; arrow shows tumor necrosis in lower left corner. Original magnification: 40×. **(f) **Higher magnification shows neoplastic cells diffusely infiltrating the stroma. Original magnification: 200×.

Mammary tumor histology data and morphology are presented in Table 2 and Fig. 2, respectively. Nine rats (32%) in the lean group developed mammary tumors compared with 17 rats (68%) in the obese group. Only two rats (7%) in the lean group had at least one tumor graded as intraductal proliferation compared with four rats (16%) in the obese group. Five rats (19%) in the lean group had at least one tumor graded as ductal carcinoma *in situ*, compared with six rats (24%) in the obese group. Obesity significantly increased (*P *< 0.05) the number of rats (7 rats, 28%) with at least one tumor graded as invasive ductal and lobular carcinoma compared with the lean group (2 rats, 7%). A total of 53 mammary tumors were detected in the study; 37 tumors (70% of the total tumors) were detected in the obese group versus 16 tumors (30% of the total tumors) in the lean group. Obesity was associated with a nonsignificant increase in tumor weight.

## Discussion

Higher body weight is associated with increased incidence of both spontaneous and chemically induced mammary tumors in various strains of mice [9,10,12,28]. For example, the *Lepr*^*db *^and *Lep*^*ob*^-TGFα transgenic mouse model, used to investigate the effects of obesity on tumorigenesis, are leptin deficient or have a leptin receptor defect. However, these mice do not develop mammary tumors and therefore are not a suitable model in which to study the role of obesity in breast cancer [29]. In the present study we observed that obese Zucker rats are more susceptible to DMBA-induced tumorigenesis than are lean rats. These data clearly demonstrate the following when DMBA was administered as a carcinogen to female Zucker rats: obese rats developed mammary tumors at a faster rate than did lean rats; obese rats exhibited shorter latency periods, both at the time of appearance of the first tumor and at the day at which tumor incidence reached 25%, than lean did rats; obesity resulted in a greater incidence of mammary tumors; obese rats developed significantly more invasive ductal and lobular carcinoma than did lean rats; and obese rats had a higher tumor multiplicity, albeit not statistically significantly so, compared with lean rats.

Using Sprague–Dawley female rats, Klurfeld and colleagues [30] studied the effects of calorie restriction on mammary tumor induction by DMBA in rats fed a high-fat diet. They found that a 25% calorie restriction resulted in a significant reduction in mammary tumor incidence and tumor weight in both control rats and rats fed a high-fat diet. Further experiments implicated decreased serum insulin and insulin-like growth factor I levels in the inhibition of mammary tumor promotion in calorie restricted rats [31].

In agreement with the present data in female Zucker rats, genetically obese LA/N-cp (corpulent) female rats developed more mammary tumors than did phenotypically lean littermates when they were treated with DMBA. Klurfeld and coworkers [28] reported that calorie restriction (40%) reduced tumor incidence to 27%, compared with the 100% tumor incidence observed in obese animals fed *ad libitum*. Those investigators suggested that calorie restriction resulted in decreased insulin levels, implicating hyperinsulinemia in obese *ad libitum *fed rats as a mechanism in increased tumor promotion.

The number of overweight and obese Americans has doubled in the past two decades, which may have an impact on cancer risk and survival [1]. The association between obesity and breast cancer risk has been inconclusive in a number of epidemiologic studies. Several studies have reported that increased body mass in premenopausal women is not associated with increased risk for breast cancer, whereas the same studies have demonstrated a positive association between increased body mass and breast cancer risk in postmenopausal women [32,33]. Recent epidemiologic studies have suggested that the age at which a woman gains weight may be a more relevant factor in determining breast cancer risk [34].

Our results are in contrast to those of a previous study, which used MNU as a carcinogen to induce mammary tumors. In that study [20] the authors reported that lean and obese rats had developed tumors at the same rate at 29 weeks after MNU treatment. However, 50% of the lean rats developed carcinomas of the mammary gland, as compared with only 10% of the obese rats. Also, they reported a higher incidence of colorectal tumors in obese rats than in lean rats. Although MNU and DMBA both produce mammary tumors in female rats, these tumors arise by different mechanisms of action [35]. MNU is a direct acting carcinogen and does not require metabolic activation in order to form adducts that damage DNA [36,37]. DMBA is a procarcinogen that requires metabolic activation by cytochrome P450 enzymes to reactive metabolites (dihydrodiolepoxides) that can form mutagenic DNA adducts [35]. These reactions are catalyzed principally by CYP1A2 in the liver and CYP1A1 and CYP1B1 in peripheral tissues such as the mammary gland.

Our findings are consistent with those reported for induction of colon cancer with azoxymethane in mature, genetically obese male Zucker rats. In that study [38] obese Zucker rats had significantly more colonic aberrant crypt formation than did any of the lean rats. Although the mechanism for an association with colon carcinogenesis is unknown, it is hypothesized that insulin and leptin resistance may play a role because leptin levels are typically threefold higher in the genetically obese Zucker (fa/fa) rats than in their lean counterparts, because of the leptin receptor defect in these animals [39]. However, recent results from epidemiological studies do not support the hypothesis that plasma leptin is a risk factor for breast cancer [40]. Nevertheless, body composition and weight are considered breast cancer risk factors that may influence prognosis [41].

The relevance of DMBA is that humans are exposed to DMBA and other polycyclic aromatic hydrocarbons through environmental or dietary sources, which may function in a synergistic manner with obesity and breast carcinogenesis. Exposure to polycyclic aromatic hydrocarbons in meats cooked at high temperature has been implicated in the development of breast cancer [42,43]. We have demonstrated that obesity increases the susceptibility of female Zucker rats to development of mammary tumors when DMBA is used as the inducing carcinogen. The mechanisms responsible for the observed increased risk for developing mammary tumors with obesity are not clearly understood. Several mechanisms may play roles in explaining the relationship we observed between obesity and increased mammary tumor development, including adipose associated hormones, adipokines, and inflammation [44], which are manifested by the general genetics of the animals; the exact relationships remain undefined and further work is needed. Future experiments with this model will focus on delineating the mechanisms responsible for this increased susceptibility to mammary cancer.

## Conclusion

In conclusion, we have demonstrated that obesity increases the susceptibility of female Zucker rats to development of mammary tumors when DMBA is used as the inducing carcinogen. The mechanisms responsible for the observed increased risk for developing mammary tumors with obesity remain to be defined.

## Abbreviations

BMI = body mass index; DMBA = 7,12-dimethylbenz(a)anthracene; MNU = *N*-methyl-*N*-nitrosourea.

## Competing interests

The author(s) declare that they have no competing interests.

## Authors' contributions

RH applied for and received funding for this project, designed and supervised the study, and participated in collection of tumor data and writing of the manuscript. AH coordinated and carried out the daily activities regarding overseeing the animals, recording data, and processing the samples. SM participated in the study design, interpretation of the results, and drafting of the manuscript. PS and CHJ participated in the study design and performed the statistical analyses. GF participated in the study design. TK-E contributed to the study design, analysis and interpretation of the results, and writing of the manuscript. SK provided expertise in classifying and analyzing all of the mammary tumors and participated in interpretation of study results and writing of the manuscript. All authors read and approved the final manuscript.

## Figures and Tables

**Table 1:** Organ weights of lean and obese female zucker rats

Organ	Lean (*n *= 28)	Obese (*n *= 25)
Liver		
Absolute	8.10 ± 0.14	22.08 ± 0.72**
Relative	3.05 ± 0.038	4.38 ± 0.12**
Kidney		
Absolute	1.62 ± 0.35	2.95 ± 0.10**
Relative	0.61 ± 0.01	0.59 ± 0.02

Organ weights (absolute weights) are given in grams; organ weight to body weight ratios are given as grams organ weight/grams body weight as a percentage (mean ± standard error). **P < 0.001 versus the lean group (Fisher's exact test).

**Table 2:** Characteristics of DMBA-induced mammary tumors in lean and obese female Zucker rats

Characteristic	Lean (*n *= 28)	Obese (*n *= 25)
Tumor onset		
Day of first tumor^a^	86	49**
Day at 25% tumors^b^	118	66**
*P*^c^		0.001
Tumor incidence		
% of rats with tumors^d^	32	68**
*P*^e^		0.001
Multiplicity^f^	1 (1–4)	2 (1–4)
*P*^c^		0.42
Tumor weight (g)^g^	0.37 (0.01–6.44)	0.42 (0.05–7.70)
*P*^c^		0.47
Rats with tumors^h^		
IDP	2 (7%)	4 (16%)
DCIS	5 (19%)	6 (24%)
IDC	2(7%)	7 (28%)*

^a^Post-DMBA (7,12-dimethylbenz(*a*)anthracene) day at which the first mammary tumor was detectable by palpation. ^b^Post-DMBA day at which the probability of tumor development was 25%. ^c^*P *values based on generalized Wilcoxon test. ^d^Percentage of rats with at least one mammary tumor. ^e^*P *values based on the Fisher's exact test. ^f^Median number of tumors in tumor bearing rats (minimum-maximum in parentheses). ^g^Median tumor weight (minimum-maximum in parentheses). ^h^Number and percentage of rats with at least one tumor graded as intraductal proliferation (IDP), ductal carcinoma *in situ *(DCIS), or invasive ductal and lobular carcinoma (IDC), as described in the Materials and method section. **P < 0.001, *P < 0.05.

## References

[B1] National Center for Health Statistics National Health and Nutrition Examination Survey. http://www.cdc.gov/nchs/nhanes.htm.

[B2] Anonymous (1998). NHLBI Obesity Education Initiative Expert Panel. Obes Res.

[B3] Mokdad AH, Ford ES, Bowman BA, Dietz WH, Vinicor F, Bales VS, Marks JS (2003). Prevalence of obesity, diabetes, and obesity-related health risk factors, 2001. JAMA.

[B4] Cleary MP, Maihle NJ (1997). The role of body mass index in the relative risk of developing premenopausal versus postmenopausal breast cancer. Proc Soc Exp Biol Med.

[B5] Willett WC (1997). Fat, energy and breast cancer. J Nutr.

[B6] Galanis DJ, Kolonel LN, Lee J, Le Marchand L (1998). Anthropometric predictors of breast cancer incidence and survival in a multi-ethnic cohort of female residents of Hawaii, United States. Cancer Causes Control.

[B7] Ballard-Barbash R, Swanson CA (1996). Body weight: estimation of risk for breast and endometrial cancers. Am J Clin Nutr.

[B8] Calle EE, Rodriguez C, Walker-Thurmond K, Thun MJ (2003). Overweight, obesity, and mortality from cancer in a prospectively studied cohort of U.S. adults. N Engl J Med.

[B9] Waxler SH, Tabar P, Melcher LR (1953). Obesity and the time of appearance of spontaneous mammary carcinoma in C3H mice. Cancer Res.

[B10] Waxler S (1960). Obesity and cancer susceptibility in mice. Am J Clin Nutr.

[B11] Haseman JK, Bourbina J, Eustis SL (1994). Effect of individual housing and other experimental design factors on tumor incidence in B6C3F1 mice. Fundam Appl Toxicol.

[B12] Wolff GL, Kodell RL, Cameron AM, Medina D (1982). Accelerated appearance of chemically induced mammary carcinomas in obese yellow (Avy/A) (BALB/c X VY) F1 hybrid mice. J Toxicol Environ Health.

[B13] Tartaglia LA, Dembski M, Weng X, Deng N, Culpepper J, Devos R, Richards GJ, Campfield LA, Clark FT, Deeds J (1995). Identification and expression cloning of a leptin receptor, OB-R. Cell.

[B14] Chua SC, Chung WK, Wu-Peng XS, Zhang Y, Liu SM, Tartaglia L, Leibel RL (1996). Phenotypes of mouse diabetes and rat fatty due to mutations in the OB (leptin) receptor. Science.

[B15] Zucker L, Zucker TF (1961). Fatty, a new mutation in the rat. J Heredity.

[B16] Zucker TF, Zucker LM (1963). Fat accretion and growth in the rat. J Nutr.

[B17] Zucker LM (1972). Fat mobilization in vitro and in vivo in the genetically obese Zucker rat 'fatty'. J Lipid Res.

[B18] Bray GA, York DA, Fisler JS (1989). Experimental obesity: a homeostatic failure due to defective nutrient stimulation of the sympathetic nervous system. Vitam Horm.

[B19] Bray GA (1977). The Zucker-fatty rat: a review. Fed Proc.

[B20] Lee WM, Lu S, Medline A, Archer MC (2001). Susceptibility of lean and obese Zucker rats to tumorigenesis induced by N-methyl-N-nitrosourea. Cancer Lett.

[B21] Hakkak R, Korourian S, Shelnutt SR, Lensing S, Ronis MJ, Badger TM (2000). Diets containing whey proteins or soy protein isolate protect against 7,12-dimethylbenz(a)anthracene-induced mammary tumors in female rats. Cancer Epidemiol Biomarkers Prev.

[B22] Hakkak R, Kourourian S, Fletcher T, Hale J, Fergusonn M, Ronis M, Badger T (2002). The effects of rice protein isolate on DMBA-induced mammary tumors in rats. Proc Am Assoc Cancer Res.

[B23] Altman DG (1991). Practical Statistics for Medical Research.

[B24] Cox DR, Oakes D (1984). Analysis of Survival Data.

[B25] Gross AJ, Clark VA (1975). Survival Distribution: Reliability Applications in Biomedical Sciences.

[B26] Bhattacharyya GK, Johnson RA (1977). Statistical Concepts and Methods.

[B27] Kruskal WH, Wallis WA (1952). Use of ranks in one-criterion variance analysis. J Am Stat Assoc.

[B28] Klurfeld DM, Lloyd LM, Welch CB, Davis MJ, Tulp OL, Kritchevsky D (1991). Reduction of enhanced mammary carcinogenesis in LA/N-cp (corpulent) rats by energy restriction. Proc Soc Exp Biol Med.

[B29] Hu X, Juneja SC, Maihle NJ, Cleary MP (2002). Leptin: a growth factor in normal and malignant breast cells and for normal mammary gland development. J Natl Cancer Inst.

[B30] Klurfeld DM, Welch CB, Lloyd LM, Kritchevsky D (1989). Inhibition of DMBA-induced mammary tumorigenesis by caloric restriction in rats fed high-fat diets. Int J Cancer.

[B31] Ruggeri BA, Klurfeld DM, Kritchevsky D, Furlanetto RW (1989). Caloric restriction and 7,12-dimethylbenz(a)anthracene-induced mammary tumor growth in rats: alterations in circulating insulin, insulin-like growth factors I and II, and epidermal growth factor. Cancer Res.

[B32] Lahmann PH, Hoffmann K, Allen N, van Gils CH, Khaw KT, Tehard B, Berrino F, Tjonneland A, Bigaard J, Olsen A (2004). Body size and breast cancer risk: findings from the European Prospective Investigation into Cancer And Nutrition (EPIC). Int J Cancer.

[B33] van den Brandt PA, Spiegelman D, Yaun SS, Adami HO, Beeson L, Folsom AR, Fraser G, Goldbohm RA, Graham S, Kushi L (2000). Pooled analysis of prospective cohort studies on height, weight, and breast cancer risk. Am J Epidemiol.

[B34] Morimoto LM, White E, Chen Z, Chlebowski RT, Hays J, Kuller L, Lopez AM, Manson J, Margolis KL, Muti PC (2002). Obesity, body size, and risk of postmenopausal breast cancer: the Women's Health Initiative (United States). Cancer Causes Control.

[B35] Rowlands JC, He L, Hakkak R, Ronis MJ, Badger TM (2001). Soy and whey proteins downregulate DMBA-induced liver and mammary gland CYP1 expression in female rats. J Nutr.

[B36] Russo J, Russo IH (1996). Experimentally induced mammary tumors in rats. Breast Cancer Res Treat.

[B37] Russo IH, Russo J (1996). Mammary gland neoplasia in long-term rodent studies. Environ Health Perspect.

[B38] Weber RV, Stein DE, Scholes J, Kral JG (2000). Obesity potentiates AOM-induced colon cancer. Dig Dis Sci.

[B39] White BD, Martin RJ (1997). Evidence for a central mechanism of obesity in the Zucker rat: role of neuropeptide Y and leptin. Proc Soc Exp Biol Med.

[B40] Stattin P, Soderberg S, Biessy C, Lenner P, Hallmans G, Kaaks R, Olsson T (2004). Plasma leptin and breast cancer risk: a prospective study in northern Sweden. Breast Cancer Res Treat.

[B41] Enger SM, Greif JM, Polikoff J, Press M (2004). Body weight correlates with mortality in early-stage breast cancer. Arch Surg.

[B42] Li D, Wang M, Dhingra K, Hittelman WN (1996). Aromatic DNA adducts in adjacent tissues of breast cancer patients: clues to breast cancer etiology. Cancer Res.

[B43] Rundle A, Tang D, Hibshoosh H, Estabrook A, Schnabel F, Cao W, Grumet S, Perera FP (2000). The relationship between genetic damage from polycyclic aromatic hydrocarbons in breast tissue and breast cancer. Carcinogenesis.

[B44] Trayhurn P, Wood IS (2004). Adipokines: Inflammation and the pleiotropic role of white adipose tissue. Br J Nutr.

